# Quick concurrent responses to global and local cognitive information underlie intuitive understanding in board-game experts

**DOI:** 10.1038/srep05894

**Published:** 2014-07-31

**Authors:** Hironori Nakatani, Yoko Yamaguchi

**Affiliations:** 1 Laboratory for Dynamics of Emergent Intelligence, RIKEN Brain Science Institute, 2-1, Hirosawa, Wako-shi, Saitama, 351-0198 Japan; 2 Current address: Center for Evolutionary Cognitive Science, Graduate School of Arts and Sciences, The University of Tokyo, Japan.; 3 Current address: Neuroinformatics Japan Center, RIKEN Brain Science Institute, Japan.

## Abstract

Experts have the superior cognitive capability of quickly understanding complex information in their domain; however, little is known about the neural processes underlying this ability. Here, using a board game named shogi (Japanese chess), we investigated the brain activity in expert players that was involved in their quick understanding of board-game patterns. The frontal area responded only to meaningful game positions, whereas the temporal area responded to both game and random positions with the same latency (200 ms). Subsequent to these quick responses, the temporal and parietal areas responded only to game positions, with a latency of 700 ms. During the responses, enhanced phase synchronization between these areas was observed. Thus, experts first responded to global cognitive information that was specific to game positions and to local cognitive information that was common to game and random positions concurrently. These types of information were integrated via neural synchronization at the posterior areas. As these properties were specific to experts, much of the experts' advantage in understanding game positions occurred within 1 s of perception.

Board games have been commonly used to investigate the mechanisms underlying cognitive expertise, such as perception, memory and problem-solving, because game skills can be measured and decomposed into their components for laboratory experiments^1^,^2^,^3^,^4^,^5^,^6^,^7^.

In an early study of chess expertise, de Groot found that world-class players rapidly accessed the best move during their initial perception of chess positions, although the prevailing view at the time was that chess masters attained their high-level performances of finding the best move via an extensive search of chess moves^1^,^2^.

Subsequent to de Groot's study, Chase and Simon proposed that expert chess players quickly perceive chess positions by chunking^3^,^4^. A chunk is long-term memory information regarding a pattern of piece positions. It consists of four or five pieces and a unit of perception. Gobet and Simon later proposed that chunks evolve into larger and more complex knowledge structures called templates because a chunk is too small to explain expert performances^8^,^9^. Unlike chunks, which are fixed patterns of piece positions, templates are variable patterns that consist of chunks and pieces. Templates are instantiated in a matter of seconds based on the functional relationships between chunks and pieces. The concept of a template is familiar in cognitive psychology and in artificial intelligence; it has also been called schema or frame^8^. The integrated representation of piece positions using a template allows experts to understand a game situation quickly and make their move.

The board game named shogi is a popular variant of the family of chess games that is native to Japan^10^. Similar to chess experts, expert shogi players understand game positions and move quickly^11^. Therefore, one of the prominent abilities of experts appears to be an “intuitive” understanding of positions and moves. “Intuitive” means that an idea of positions and moves comes to mind quickly, without reasoning. Using shogi, we aimed at investigating the neural processes that underlie the “intuitive” understanding of game positions. We were especially interested in the temporal properties of these neural processes, to evaluate the speed of “intuitive” understanding.

Recent neuroimaging studies about chess and shogi expertise reported that the parietal and temporal brain areas are responsible for the perception of game positions in expert players. Some regions show larger activation for meaningful game positions than they do for random positions^11^,^12^,^13^,^14^,^15^,^16^,^17^; these are the precuneus^11^, parahippocampal gyrus^12^, collateral sulci^13^, retrosplenial cortex^14^, posterior cingulate^15^ and insula^16^. Other regions respond to both game and random positions similarly; these are the posterior middle temporal gyrus, occipitotemporal junction^13^ and fusiform face area^17^.

Game positions have cognitive information that is associated with the shogi game, such as game situations or moves. The cognitive information is represented by templates^8^,^9^. In contrast, random positions do not have such information, but share local features, i.e., pieces, with game positions. Each piece also has cognitive information such as its roles and values in the shogi game. There are two types of neural processes that underlie the understanding of game positions: one is responsible for the global cognitive information that is specific to game positions, whereas the other is responsible for the local cognitive information that is common to game and random positions.

The nature of the relationship between these two types of neural processes remains unknown. It is possible that game positions are analysed serially, from local to global cognitive information. This view is consistent with the traditional view that visual input is processed serially, from simple features to increasingly complex information. A different view is also possible. In game positions, chunks and pieces have functional relationships with each other to form global cognitive information. The perception of a salient part of these relationships will lead to the quick capture of the conceptual gist of global cognitive information, without recognition of the details of individual local cognitive information. If this is the case in experts, the neural process that is responsible for global cognitive information should appear at the early stage of game-position understanding.

To investigate the spatio-temporal dynamics of the neural processes underlying game-position understanding, we measured electroencephalogram (EEG) in shogi players who responded to the presentation of game and random positions. We found that quick and concurrent processes of global and local cognitive information mediated the “intuitive” understanding of game positions in experts.

## Results

### Experimental design and participants

**Figure 1:**
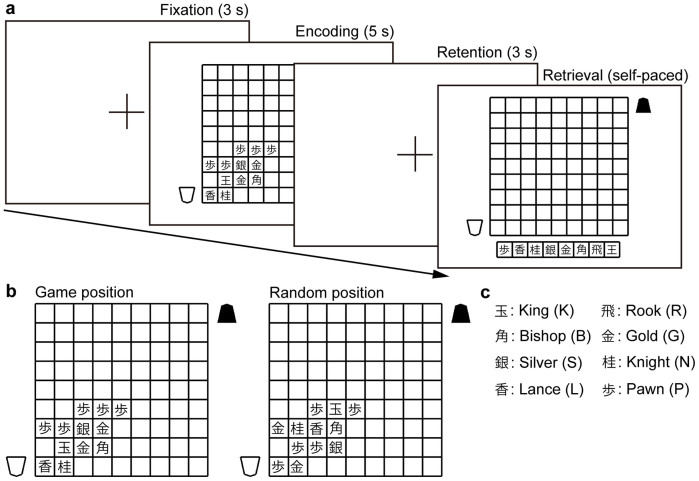
Experimental design. (a) Visual short-term memory task using shogi pieces. In each trial, the participants memorized a piece position that was presented in the encoding phase. Game positions or random positions were presented in randomized order. After the retention phase, a shogi board and pieces were presented on the monitor. Each piece could be placed on the board using a computer mouse. The participants reconstructed the presented piece positions from memory. (b) Left panel: example of the game positions used in the experiment. We used defence or attack formations in an opening of a shogi game as stimuli in the game condition. In this example, the game position represents a defence formation called *Yagura*. Right panel: example of the random positions used in the experiment. The random positions were created from a random rearrangement of pieces in the game condition. The number and type of pieces and occupied squares in the stimuli of the random condition were, therefore, the same as the corresponding stimuli of the game condition. (c) Eight Japanese characters and their English names used to identify the types of pieces.

We carried out short-term memory experiments for shogi piece positions (Fig. 1a). For each trial of experiments, the participants memorized a piece position that was presented visually during a 5 s encoding phase. Each position consisted of an average of 12.1 pieces. After a 3 s retention phase, a shogi board and pieces were presented on the monitor. Each piece could be placed on the board using a computer mouse. The participants reconstructed piece positions from memory. There were two stimulus conditions (Fig. 1b). One was a game condition where piece positions were taken from game records. Each game position represented a defence or an attack formation in shogi opening. The other was a random condition where the piece positions were created by the random rearrangement of the pieces among the positions occupied in the game condition. The number and type of pieces and the squares occupied in the random-position stimuli were, therefore, the same as in the corresponding stimuli in the game positions. None of the random positions was associated with shogi strategy. Japanese characters were used to identify the eight types of pieces (Fig. 1c).

**Figure 2:**
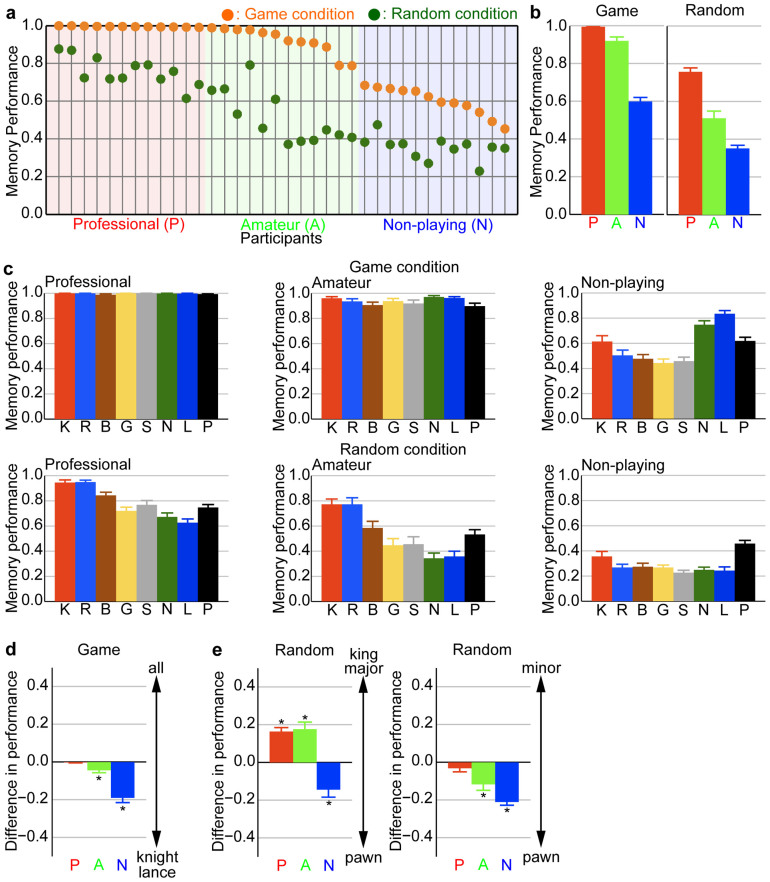
Memory performances and properties varied according to shogi expertise. (a) Memory performances of the individual participants in the game and random conditions. Participants were sorted from left to right on the horizontal axis based on their memory performances in the game condition. They were classified into three groups. The high-performance group consisted only of professional players (*n* = 12), the middle-performance group consisted only of amateur players (*n* = 12), and the low-performance group consisted only of non-playing individuals (*n* = 12). (b) Group averages of memory performance in the game and random conditions. P, professional group; A, amateur group; and N, non-playing group. Error bars indicate the standard error across participants within the group. (c) Group averages of memory performance for each type of shogi piece in the game and random conditions. K, king; R, rook; B, bishop; G, gold; S, silver; N, knight; L, lance; and P, pawn. The error bars indicate the standard error across participants within the group. (d) Comparison between memory performances regarding all pieces vs the knight and lance in the game condition. The knight and lance tend to be located at their initial positions as a pair in an opening of a shogi game. (e) Left panel: comparison between memory performances regarding the king and major pieces (the rook and the bishop) vs the pawn in the random condition. The king is the most important piece in a shogi game. The major pieces are the most powerful pieces; therefore, they are strategically salient. The pawn is the least powerful piece. As about half of the pieces are pawns, the pawn is perceptually salient. Right panel: comparison between memory performances regarding the minor pieces (the gold, silver, knight and lance) vs the pawn in the random condition. The minor pieces are less powerful than the major pieces in a shogi game. The error bars indicate the standard error across participants within the group. The asterisks denote significant differences (*n* = 12 for each group, *P* < 0.01; two-tailed *t* test).

This study included 36 participants, including professional players (*n* = 12), amateur players (*n* = 12) and non-playing individuals (*n* = 12). The professional players were members of the Japan Shogi Association. We first evaluated the shogi expertise of each participant using behavioural measures. According to chess studies, memory performance regarding chess positions is a good measure of chess skill^3^,^5^,^6^,^18^,^19^,^20^. We sorted participants according to memory performance regarding game positions and divided them into three groups (Fig. 2a). The high-, middle- and low-performance groups consisted only of professional players, amateur players and non-playing individuals, respectively. Thus, in this study, all professional players had higher shogi expertise than did amateur players and non-playing individuals. At post-experiment interviews, both professional and amateur players were able to distinguish the game positions from the random positions used in this study; in contrast, non-playing individuals were not.

### Shogi game knowledge reflects memory properties in experts

Our analysis of behavioural data showed that professional players took advantage of their knowledge about global and local cognitive information associated with the shogi game to perform our experiment. Their knowledge of global cognitive information was applicable in a more natural way to the recognition of game positions than it was to the recognition of random positions. Professional players memorized game positions better than they did random positions (*t_11_* = 10.97, *P* < 0.0001; two-tailed paired *t* test; note that when the actual *P* value was <0.0001, we denoted it as *P* < 0.0001 as the actual *P* value throughout the text; Fig. 2b). Amateur players also memorized game positions better compared with random positions (*t_11_* = 13.20, *P* < 0.0001; two-tailed paired *t* test; Fig. 2b).

Next, we focused on the knowledge about local cognitive information. In the shogi game, different types of pieces have different roles and values. We defined three categories of pieces. The first category included the king and major pieces (the rook and the bishop). The king is the most important piece in a shogi game. The major pieces are the most powerful pieces; hence, they are strategically salient. The second category included the minor pieces (the gold general, the silver general, the knight and the lance); these are less powerful than the major pieces. The third category included the pawn, which is the least powerful piece. However, as about half of the pieces are pawns, the pawn is perceptually salient. There seemed to be a memory preference regarding the type of pieces in the random condition (Fig. 2c). We compared memory performances between strategically and perceptually salient pieces: the first category vs the third category and the second category vs the third category. Memory properties varied according to shogi expertise. Groups with higher expertise memorized strategically salient pieces preferentially (*F_1,66_* = 38.97, *P* < 0.0001; two-way analysis of variance (ANOVA), main effect of group; Fig. 2e). The non-playing group showed no differences between the first and the second categories (*t_11_* = 1.86, *P* = 0.0898; two-tailed paired *t* test), which indicated that the strategic salience of pieces in the shogi game did not affect the memory properties of this group. We noted that groups with higher expertise showed not only strategic salience-based memory preference, but also better memory performances, even in the random condition (*F_2,33_* = 52.55, *P* < 0.0001; one-way ANOVA; Fig. 2b).

Non-playing individuals also showed better memory performances for game positions than they did for random positions (*t_11_* = 8.89, *P* < 0.0001; two-tailed paired *t* test; Fig. 2b). In the game condition, they memorized preferentially two types of pieces, the knight and the lance (*t_11_* = 7.04, *P* < 0.0001; two-tailed paired *t* test; comparison of memory performances between the knight and the lance vs all pieces in the game condition; Fig. 2c,d). In the opening of a shogi game, these two pieces tend to be located at their initial positions. In our experiments, these pieces were placed at their initial positions as a pair in 36 out of 50 trials in the game condition. Based on these physical stimuli properties, non-playing individuals might recognize the knight and the lance as a chunk. In the random condition, the non-playing group memorized the perceptually salient piece, the pawn, preferentially (*t_11_* = −3.57, *P* = 0.0022 for the first category vs the third category of pieces; *t_11_* = −11.08, *P* < 0.0001 for the second category vs the third category of pieces; Fig. 2e).

The memory properties of professional players reflected their knowledge of shogi strategy. Conversely, the memory properties of non-playing individuals reflected the physical stimuli properties.

### Experts' quick EEG responses to the presentation of game positions

**Figure 3:**
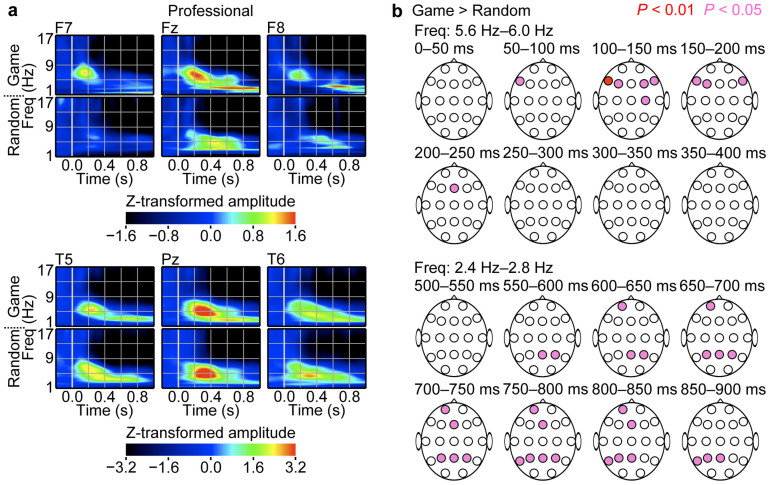
The frontal areas of the brains of the individuals in the professional group responded to game positions quickly. (a) EEG responses recorded in the professional group. The group averages of time-frequency representations of the EEG responses to the stimulus presentations at the encoding phase are presented. The amplitude of the EEG responses in the time-frequency domain was Z transformed, aligned with the stimulus onset and averaged across trials. The top and bottom panels represent EEG responses in the game and random conditions, respectively. (b) The topographies of significant differences in the amplitude of the EEG responses of the professional group between the game and random conditions are presented (*n* = 12; two-tailed paired *t* test). Time 0 ms indicates the timing of piece-position presentation. The colour red denotes a larger amplitude in the game condition compared with the random condition. A larger amplitude in the random condition was not observed.

**Figure 4:**
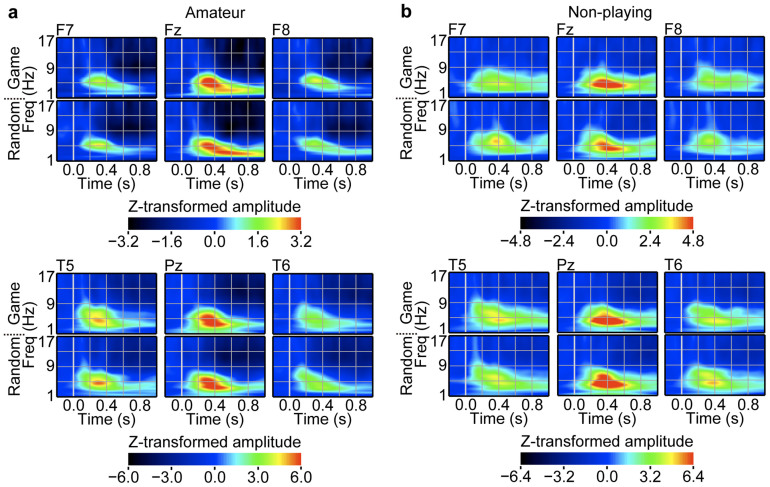
Non-experts showed similar responses to game and random positions. Group averages of time-frequency representations of the EEG responses to the stimulus presentations at the encoding phase are presented. The amplitude of the EEG responses in the time-frequency domain was Z transformed, aligned with the stimulus onset and averaged across trials. The top and bottom panels represent EEG responses in the game and random conditions, respectively. (a) EEG responses of the amateur group. (b) EEG responses of the non-playing group.

To investigate the neural process underlying the understanding of game positions in experts, we analysed EEG responses to the presentation of piece positions at the encoding phase. Figures 3a and 4a,b show examples of time-frequency representations of the EEG amplitude responses at the frontal, temporal and parietal areas in the game and random conditions. As the EEG responses were located mainly at the delta and theta bands (below 8 Hz), we focused our analyses on these frequency components.

**Figure 5:**
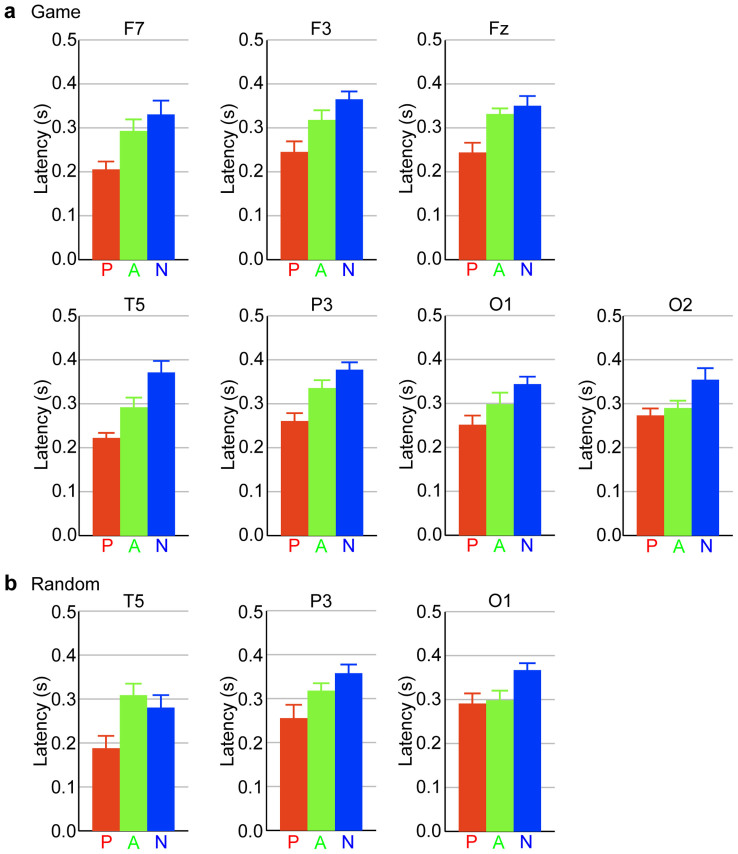
The frontal and left posterior brain areas responded to piece positions faster with increasing expertise. Group averages of response latencies at recording sites that showed expertise-related differences are presented. (a) Game condition. (*F_2,33_* = 4.305, *P* = 0.0218 for F7; *F_2,33_* = 5.432, *P* = 0.0091 for F3; *F_2,33_* = 5.740, *P* = 0.0073 for Fz; *F_2,33_* = 10.403, *P* = 0.0003 for T5; *F_2,33_* = 9.219, *P* = 0.0007 for P3; *F_2,33_* = 4.119, *P* = 0.0253 for O1; *F_2,33_* = 4.026, *P* = 0.0273 for O2; one-way ANOVA). (b) Random condition (*F_2,33_* = 4.723, *P* = 0.0157 for T5; *F_2,33_* = 4.573, *P* = 0.0177 for P3; *F_2,33_* = 4.135, *P* = 0.0250 for O1; one-way ANOVA). The error bars indicate the standard error within the group.

First, we sought to identify EEG responses that were associated with global cognitive information specific to game positions. We compared the EEG responses of professional players to game positions with their responses to random positions. The professional group showed game-position-specific responses in the theta and delta bands (e.g., *t_11_* = 3.22, *P* = 0.0041 for F7 100–150 ms; *t_11_* = 2.55, *P* = 0.0136 for Fz 200–250 ms; *t_11_* = 2.60, *P* = 0.0123 for Pz 600–650 ms; *t_11_* = 2.44, *P* = 0.0165 for Fz 750–800 ms; two-tailed paired *t* test; Fig. 3b). Transient periods of amplitude increase in the theta band first appeared at the left and right sides of the lateral frontal areas, with a relatively short latency of 100–200 ms, which was followed by a frontal midline response (Fig. 3b). In addition, the delta band response followed the theta band response; it appeared at the parietal, temporal and frontal areas with a longer latency of 600–900 ms (Fig. 3b). The amateur and non-playing groups did not show such game-position-specific responses; however, because these groups also showed frontal responses to game positions (Fig. 4a,b), we investigated whether frontal responses exhibited expertise-related properties. Frontal responses to game positions were faster as a function of expertise (*F_2,33_* = 4.305, *P* = 0.0218 for F7; *F_2,33_* = 5.432, *P* = 0.0091 for F3; *F_2,33_* = 5.740, *P* = 0.0073 for Fz; one-way ANOVA; Fig. 5a).

Next, we identified the EEG responses that were associated with local cognitive information. As local cognitive information existed in game and random positions, local cognitive information-related EEG responses were expected in both types of positions similarly, with expertise-related properties. The professional group exhibited faster responses at the left posterior areas to game and random positions similarly than did the amateur and non-playing groups (in game conditions: *F_2,33_* = 10.403, *P* = 0.0003 for T5; *F_2,33_* = 9.219, *P* = 0.0007 for P3; *F_2,33_* = 4.119, *P* = 0.0253 for O1; one-way ANOVA; Fig. 5a; in random conditions: *F_2,33_* = 4.723, *P* = 0.0157 for T5; *F_2,33_* = 4.573, *P* = 0.0177 for P3; *F_2,33_* = 4.135, *P* = 0.0250 for O1; one-way ANOVA; Fig. 5b).

If game positions are analysed serially from simple local to increasingly complex global cognitive information, then the temporal response to local cognitive information should precede the frontal response to global cognitive information. However, there was no statistical difference in latency between the left temporal response (T5 site) and the left frontal response (F7 site) of professional players in the game condition (*t_11_* = −0.22, *P* = 0.8300; two-tailed paired *t* test; Fig. 5a), which suggests that global and local cognitive information was analysed concurrently, about 200 ms after the game-position presentation.

### Functional connectivity during EEG responses to game positions

**Figure 6:**
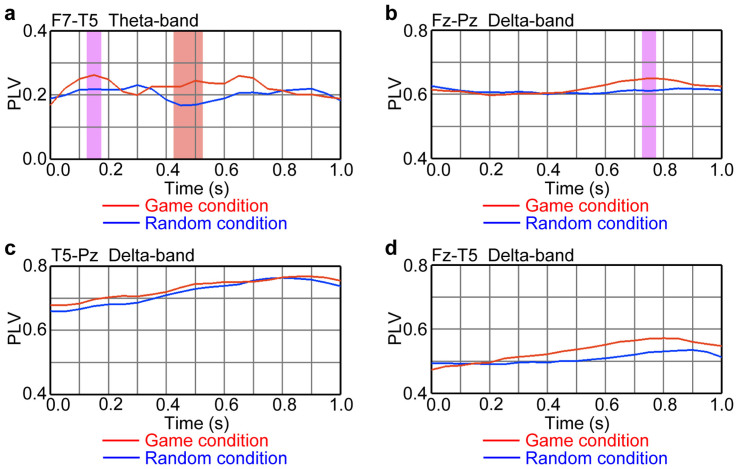
Frontal activity interacted with parietal and temporal activities in the game condition. Phase-locking between two EEG signals at the theta band (6.0 Hz) and delta band (2.4 Hz) in the professional group was evaluated based on phase-locking value (PLV). The light red and light purple colours, respectively, indicate significant (*P* < 0.05) and marginal (*P* < 0.10) differences between the game (red) and the random (blue) conditions. Time 0.0 is the timing of piece-position presentation. (a) Time series of PLVs between the left frontal activity (F7 site) and the left temporal activity (T5 site) at the theta band. (b) Time series of PLVs between the frontal activity (Fz site) and the parietal activity (Pz site) at the delta band. (c) Time series of PLVs between the left temporal activity (T5 site) and the parietal activity (Pz site) at the delta band. (d) Time series of PLVs between the frontal activity (Fz site) and the left temporal activity (T5 site) at the delta band.

The separately analysed global and local cognitive information must be integrated into meaningful information to understand the details of game positions. There should be neural communication between distant brain areas during EEG responses to game positions. Phase synchrony, i.e., phase-locking between neural activities over a limited period, is a candidate mechanism for neural communication^21^. We calculated the phase-locking value (PLV) between two EEG signals^22^,^23^. We first evaluated phase-locking between the quick and concurrent frontal–temporal responses. The PLV between the left frontal response (F7 site) and the left temporal response (T5 site) at the theta band was marginally larger in the game than in the random condition at the time at which players responded to game positions (*t_11_* = 1.89, *P* = 0.0854, marginally significant for 0.15 s after piece-position presentation; two-tailed paired *t* test; Fig. 6a).

Next, we evaluated phase-locking at the delta band during subsequent parietal, temporal and frontal responses to game positions with a latency of around 700 ms. The PLV between the frontal (Fz site) and parietal (Pz site) areas was marginally larger in the game positions than it was in the random condition (*t_11_* = 1.80, *P* = 0.0991, marginally significant for 0.75 s after piece-position presentation; two-tailed paired *t* test; Fig. 6b). The PLV between the temporal (T5 site) and parietal areas increased after piece-position presentation in the game and random conditions (*t_11_* = 6.23, *P* < 0.0001 in the game condition; *t_11_* = 4.36, *P* = 0.0011 in the random condition, for the 0.75 s that followed piece-position presentation compared with 0.0 s; two-tailed paired *t* test; Fig. 6c). The PLV between the frontal and temporal areas increased after piece-position presentation only in the game condition (*t_11_* = 3.78, *P* < 0.0036 in the game condition; *t_11_* = 0.99, *P* = 0.3455 in the random condition; two-tailed paired *t* test; Fig. 6d). Therefore, the frontal activity interacted with the parietal and temporal activities only in the game condition, whereas the parietal and temporal activities interacted with each other in the game and random conditions.

### Similar neural mechanisms contribute to memory retention of game and random positions

**Figure 7:**
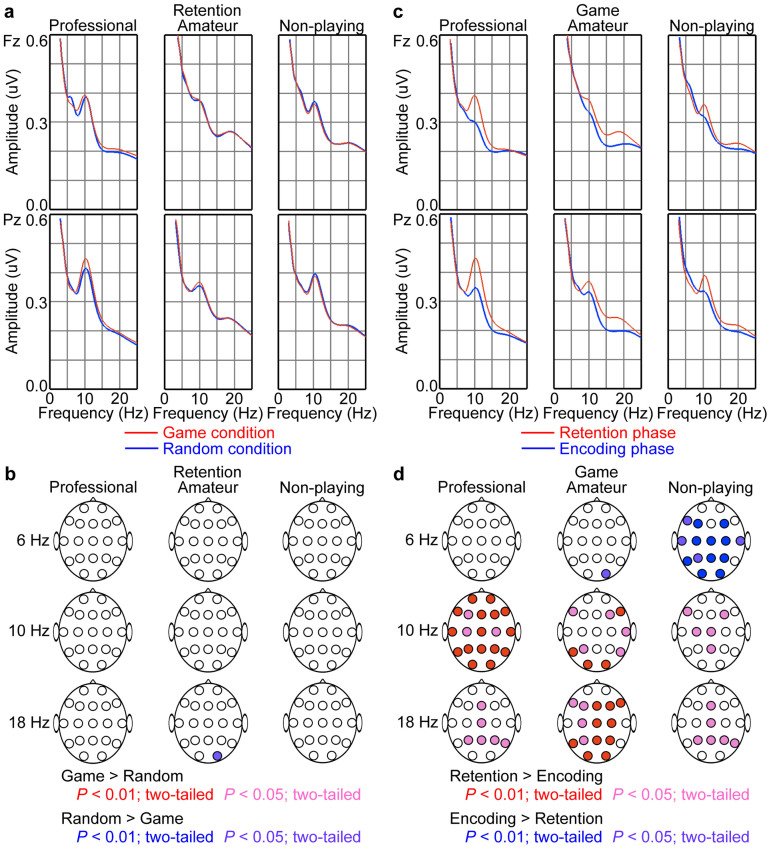
Distinct properties of the EEG spectra characterized the levels of expertise. (a) Group averages of the EEG amplitude spectra at the frontal midline area (Fz) and at the parietal midline area (Pz) during the retention phase in the game (red) and random (blue) conditions. (b) Topographies of significant differences in the spectra during the retention phase between the game and random conditions (*n* = 12 for each group; two-tailed paired *t* test). The red and blue colours denote the presence of a larger amplitude in the game and random conditions, respectively. (c) Group averages of the EEG amplitude spectra at the frontal midline area and at the parietal midline area during the retention (red) and encoding (blue) phases in the game condition. (d) Topographies of significant differences in the spectra in the game condition between the retention and encoding phases (*n* = 12 for each group; two-tailed paired *t* test). The red and blue colours denote the presence of a larger amplitude in the retention and encoding phases, respectively.

The different EEG responses to the presentation of game and random positions suggest that the neural processes used to encode piece positions were different between the game and random positions. We wondered whether the neural processes used for the maintenance of the encoded mental representation of piece positions were also different between game and random conditions. We compared EEG amplitude spectra during memory retention between game and random conditions. No significant differences were found in EEG spectra between the two conditions (Fig. 7a,b), which suggests that similar neural mechanisms contribute to the maintenance of the mental representation of game and random positions.

We found expertise-related differences in EEG spectra between the encoding and retention phases (Fig. 7c,d). The professional group showed increased activity mainly in the alpha band during the retention phase (e.g., *t_11_* = 3.23, *P* < 0.0040 for Pz at 10 Hz, *n* = 12; two-tailed paired *t* test). In contrast, the amateur and non-playing groups showed increased beta band activity and decreased theta band activity, respectively, during the retention phase (in the amateur group: e.g., *t_11_* = 4.03, *P* = 0.0010 for Pz at 18 Hz; in the non-playing group: *t_11_* = −3.62, *P* = 0.0020 for Pz at 6 Hz, *n* = 12 for each group; two-tailed paired *t* test). Thus, the neural mechanism involved in the maintenance of piece positions in short-term memory appears to depend on the level of expertise.

## Discussion

Two periods of EEG responses to the game-position presentation were specific to professional players. In the first period of the response, with a latency of 200 ms, the frontal area responded only to game positions and the left temporal area responded to both game and random positions. In the second period of the response, with a latency of 700 ms, the parietal, temporal and frontal areas responded only to game positions. These distributed brain areas showed enhanced phase synchronization during the responses to game positions. These results suggest that global and local cognitive information were analysed quickly and concurrently and were then integrated to understand the details of game positions. As these properties were specific to professional players, much of the experts' advantage in understanding game positions occurred within 1 s of perception; this quick property fits the concept of “intuitive” understanding (i.e., without reasoning).

We defined that global cognitive information was specific to game positions, and that local cognitive information was common to game and random positions. Based on template theory^8^,^9^, we considered that global cognitive information was represented by templates and that local cognitive information was represented by individual pieces. According to template theory, the integrated representations of piece positions based on structured knowledge of shogi enhance memory performances. This explains the observation that participants showed better memory performances in the game condition than they did in the random condition, and that participants with higher expertise showed better performances in the game condition. Template theory also explains the observation that experts showed better performances even in the random condition^8^. The building blocks of a template are chunks and pieces, and the template is instantiated based on the functional relationships between chunks and pieces. Random positions simply do not form a rich template because they lack chunks. However, it is possible that there are adventitious functional relationships among pieces, such as the king being in check, and that participants with higher expertise detect such relationships with higher probability than do participants with lower expertise. The similar EEG amplitude spectra observed during memory retention between game and random conditions suggest that similar cognitive mechanisms underlie the representation of game and random positions. If this is the case, the response of the left temporal area to both game and random positions reflects the recognition not only of individual pieces, but also of functional relationships among pieces. Therefore, the temporal area is responsible for the detailed pattern recognition of piece positions. This interpretation is consistent with the results of previous neuroimaging studies of chess^12^,^13^,^14^,^15^,^16^,^17^.

Our main finding was that the frontal area of professional shogi players quickly responded to game positions, concurrently with the temporal area. The frontal area is involved in visual object recognition^24^,^25^,^26^,^27^,^28^. For example, the lateral prefrontal cortex represents categories of visual objects^26^. The event-related potentials that occur at the frontal area respond to a go/no-go categorization task with a latency of 150 ms^25^, and the left orbitofrontal cortex responds to the low-spatial-frequency components of visual images with a latency of 130 ms^27^. The magnocellular pathway is a possible anatomical basis of the quick frontal response to visual input. This pathway is one of the major subdivisions of the visual pathway, the other subdivision being the parvocellular pathway. The magnocellular pathway quickly conveys low-spatial-frequency components of visual information compared with the parvocellular pathway^29^. The magnocellular-biased stimuli activate the orbitofrontal cortex more than do the parvocellular-biased stimuli, which activate the ventral occipitotemporal cortex more than do the magnocellular-biased stimuli^28^. Objects in the same category look similar to each other, whereas objects in different categories look different. Spatially coarse information would be sufficient for categorization^30^. Therefore, at the early stage of visual recognition, the frontal area might represent a coarse and holistic aspect of visual information, whereas the ventral temporal area represents the details of each of the elements of visual information.

A quick frontal response to visual input has been proposed to facilitate visual recognition at the temporal area^27^,^30^. A coarse and holistic representation of visual input activates object-related semantic knowledge in the ventral prefrontal area to generate the most likely interpretation. The holistic representation guides the integration of relevant elements into a coherent representation of visual input in the recognition-related regions within the temporal area. In the case of elements that exhibit ambiguity regarding meaning and relationship with others, the holistic representation is essential to integrate them into a coherent representation^31^.

We propose a possible scenario of neural processes underlying the quick understanding of game positions. The presentation of a game position leads to the concurrent transmission of visual information to the frontal and the ventral temporal areas by the magnocellular and parvocellular pathways, respectively, with a latency of 200 ms. In the frontal area, key features of game positions quickly activate piece-pattern-related semantic knowledge to generate the most likely interpretation of global cognitive information. Random positions do not activate semantic knowledge because they have no features associated with global cognitive information and therefore evoke no frontal responses. At the same time, the details of local cognitive information associated with individual pieces are analysed in the ventral temporal area. After the concurrent processes at the frontal and ventral temporal areas, the global and local cognitive information are integrated into a comprehensible representation of a game position, which enables the generation of an idea of game situations or moves. The second period of game-position-specific responses in the temporal, parietal and frontal areas and enhanced phase synchronization between these areas with a latency of 700 ms is a candidate process for the integration of information. Our interpretation of the second period of the responses is consistent with neuroimaging studies of chess and shogi expertise that reported that the temporal and parietal areas are responsible for the perception of game positions^11^,^12^,^13^,^14^,^15^,^16^,^17^. In the case of shogi expertise, for example, the precuneus of the parietal area is responsible for the perception of a game position. It is also responsible for the generation of an idea of moves^11^, together with the caudate nucleus of the basal ganglia. Therefore, the game-position-specific response at the parietal area may be responsible for a comprehensible representation of a game position.

Shogi expertise may share its neural mechanism with expertise in other domains. For example, the ability to understand visual words is not innate. Similar to shogi expertise, this skill is acquired through training. A study that used positron-emission tomography found that the visual word form area located in the left temporal area shows activation for both actual and pseudo words, whereas the frontal area shows activation only for actual words^32^. An EEG study reported a spatio-temporal pattern of neural processes underlying actual word recognition. Both the frontal and left temporal areas responded to visually presented actual words with a latency of 200 ms, and the posterior area responded to these words with a latency of 600–800 ms^33^. The neural processes underlying word recognition have similar properties to those underlying shogi position recognition.

Moreover, the neural processes underlying facial-expression recognition exhibit properties that are similar to those that underlie shogi position recognition. Both the frontal and lateral temporal areas respond quickly to emotional faces within 200 ms of stimulus presentation^34^,^35^. These areas respond to different aspects of faces. The frontal area responds to emotional facial expressions^35^, whereas the temporal area responds to face parts and configuration^35^,^36^. Emotional facial expression also evokes a second period of response at the posterior area, with a latency of 455–1000 ms^34^,^35^. Emotional facial expression represents cognitive information that is associated with an emotional state such as happy or sad states. This can be considered as a global aspect of the cognitive information of a face. Conversely, facial parts and configuration are features that are used to recognize a face. They can be considered as local aspects of the cognitive information of a face.

The similarity in the neural processes involved in shogi and other domains suggests that a quick, concurrent response to global and local cognitive information is not a privilege of professional shogi players. Rather, it might be a general property of cognitive function to understand visual information efficiently based on experience. In chess, around 10 years of intensive training is necessary to attain an international level of chess skills^5^. This 10-year rule can be generalized to other domains of expertise^5^. Therefore, the intensive, long-term training of professional shogi players allows them to use the general property of experience-based cognitive function for the “intuitive” understanding of a piece position in a shogi game.

## Methods

### Participants

Thirty-six participants with normal or corrected-to-normal vision took part in this study. The mean age was 31.6 years (range, 20–51 years). Twelve participants were professional players and members of the Japan Shogi Association, and the other participants were amateur players (*n* = 12) or non-playing individuals (*n* = 12). Participants gave written informed consent before the experiment. The Research Ethics Committee of RIKEN approved our procedures.

In shogi, the so-called *dan/kyu* grade-ranking system is used to indicate playing strength; this is the same terminology used for many other arts in Japan. The professional and amateur players are rated using different scales. The playing strength of the professional players in our experiments was professional 4 *dan* and above. The strength of the amateur players was between amateur 2 *kyu* and 5 *dan*, meaning that our amateur players understood the shogi strategy. The non-playing individuals were naïve about the shogi strategy, although some of them knew the rules of shogi, such as the movements of pieces and the objective of enforcing capture (checkmate) of the opponent's king.

### Experimental design

We conducted a short-term memory experiment for shogi positions (Fig. 1a). Each trial consisted of four phases: a 3 s fixation phase, a 5 s encoding phase, a 3 s retention phase and a self-paced retrieval phase. Stimuli were black drawings on a white background. The stimuli were presented visually at eye level on a flat-panel monitor with reduced ceiling illumination. Participants were seated comfortably at a distance of 80 cm from the monitor. A chin rest was used to suppress head movement.

In the fixation phase, participants fixated a cross that was located at the centre of the monitor. In the encoding phase, a stimulus was presented. Stimuli consisted of 10–15 shogi pieces and a shogi board. Two types of piece positions were used. One was a game position (Fig. 1b), in which piece positions were used from the records of shogi games of professional players. Each game position represented a defence or an attack formation at the opening of a shogi game. The other was a random position (Fig. 1b), in which piece positions were created from the random replacement of pieces in the game positions. The number and type of pieces and occupied squares in the stimuli of the random positions were the same as the corresponding stimuli of the game positions. None of the random positions was associated with shogi strategy. The stimuli were presented on an area of a shogi board that was composed of rectangles in a grid of nine ranks (rows) by nine files (columns). The board subtended the visual angle by 10° and each stimulus was typically located within 5° of the visual angle. In the retention phase, a fixation cross was located at the centre of the monitor. In the retrieval phase, a shogi board and shogi pieces were presented. Each piece could be placed on the board using a computer mouse. The participants attempted to reconstruct from memory the piece positions that had been presented previously.

We used 50 game positions and 50 random positions as stimuli. The stimuli were presented in randomized order. We performed five sessions of experiments. Each session consisted of 20 trials: 10 game trials and 10 random trials. Each session typically took 15–20 min to complete. There was a short break after each session.

### EEG measurements

We placed 19 disk-type Ag/AgCl electrodes on the scalp of each participant, in accordance with the international 10/20 system^37^. Reference and ground electrodes were placed on the left ear and forehead, respectively. Vertical and horizontal electrooculograms were also recorded. Signals were amplified and band limited using differential amplifiers and band-pass filters (SynAmps, NeuroScan, Charlotte, USA). Gains were 60 dB and cut-off frequencies were 0.1 and 100 Hz. The input impedance of the amplifiers was 10 MΩ. Signals were digitized using a 16-bit analogue-to-digital converter at 500 Hz and stored on a hard disk.

### EEG analysis

We used an independent component analysis^38^ to reduce eye-movement-related artefacts in EEG recordings. Using the FastICA algorithm^39^, we decomposed the recordings and reconstructed them after the removal of components that contained eye-movement-related artefacts.

To analyse the EEG activity in the time-frequency domain, we produced a scalogram using a continuous wavelet transform. The scalogram is the amplitude of the signal in the time-frequency domain. The mother function of the wavelet transform was the complex Gabor function, *g*(*t*): 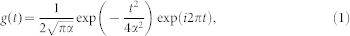
where *α* = 0.5. The wavelet coefficients of a signal, *x*(*t*), were obtained as follows: 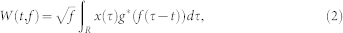
where g^*^ is the complex conjugate of a complex Gabor function. The wavelet coefficient, *W*(*t*,*f*), consists of an amplitude value |*W*(*t*,*f*)| and a phase value *φ*(*t*,*f*), such that: 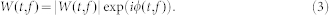
For the analysis of the EEG responses to the stimulus presentations, the amplitude |*W*(*t*,*f*)| was Z transformed, aligned with the stimulus presentations and then averaged across trials per participant.

To evaluate phase-locking between two EEG signals across trials, we calculated the PLV, which is defined as follows^22^,^23^: 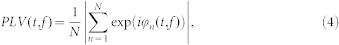
where *ϕ_n_*(*t*,*f*) is the phase difference between two signals, *φ*_1,*n*_(*t*,*f*) − *φ*_2,*n*_(*t*,*f*), at the *n*-th trial and *N* is the number of trials.

During the analysis of the EEG amplitude spectrum, we averaged the scalogram across the time domain. We calculated the spectra for the encoding phase and the retention phase separately.

## Author Contributions

H.N. and Y.Y. designed the research, H.N. and Y.Y. performed the research, H.N. analysed the data and H.N. and Y.Y. wrote the paper.
